# Student and educator perspectives on clinical reasoning: A qualitative study

**DOI:** 10.4102/sajp.v81i1.2161

**Published:** 2025-05-28

**Authors:** Danelle Hess, Jacqueline Hendricks, José Frantz, Michael Rowe

**Affiliations:** 1 Department of Physiotherapy, Faculty of Community and Health Sciences, University of the Western Cape, Cape Town, South Africa; 2 Office of the Deputy Vice Chancellor: Research and Innovation, University of the Western Cape, Cape Town, South Africa

**Keywords:** clinical reasoning, physiotherapy education, undergraduate teaching, undergraduate learning, physiotherapy, physiotherapy lecturers, physiotherapy clinical educators

## Abstract

**Background:**

When students and educators understand a skill like clinical reasoning (CR) differently, attempting to develop it becomes challenging. Miscommunication in how different stakeholders understand this essential skill can potentially harm patients.

**Objectives:**

Our study explores how physiotherapy students and educators (both lecturers and clinical educators [CEs]) in a physiotherapy department understand CR. The research aimed to identify any potential gaps in the stakeholders’ understanding of CR and explore strategies for better alignment.

**Method:**

A qualitative exploratory descriptive design was employed. In-depth interviews were conducted with 27 undergraduate physiotherapy students, 10 physiotherapy lecturers, and 8 CEs.

**Results:**

Thematic analysis revealed three key themes: cognitive process, evidence-based practice, and clinical approach. Significant differences emerged between experts (lecturers and CEs) and novices (students) in conceptualising CR. The experts demonstrated a more holistic understanding, focusing on hypothesis generation and interconnected reasoning. In contrast, students focus on information collecting and justification of actions.

**Conclusion:**

The findings highlight a gap in CR understanding that could potentially impede reaching expected learning outcomes.

**Clinical implications:**

Our study recommends seeking alignment of students’ and educators’ perspectives through structured dialogue and intentionally designed educational strategies. This includes developing holistic assessment rubrics that acknowledge both foundational and advanced CR skills and implementing case-based learning approaches. And creating opportunities for educators to make their reasoning processes explicit and visible to students.

## Introduction

Clinical reasoning (CR) skills are a cornerstone competency in physiotherapy education and physiotherapy practice, which is essential for ensuring patient safety and optimal patient outcomes (Abrandt Dahlgren et al. 2021). While numerous scholars have defined CR in physiotherapy, a recent conceptualisation by Oostendorp, Hans Elvers and Van Trijffel (2020) describes it as an iterative process in which the physiotherapist collaborates with the patient and relevant stakeholders to determine an appropriate management plan, integrating both clinical expertise and the patient’s values, goals, and expectations. Edwards et al. (2004) highlighted that CR in physiotherapy was not just focused on diagnostics and the acquisition of biomedical knowledge but also related to the problem-solving and decision-making nature of the profession, which depends on the types of patients seen and various disciplines within physiotherapy. In some disciplines of physiotherapy, such as neuromusculoskeletal physiotherapy, the diagnosis is a big part of the clinical reasoning process, whereas in disciplines such as respiratory and neurology, there is less focus on diagnosing the patient but on rather problem-solving and creating an effective management plan for the patient. The complexity of developing CR skills in health professions education lies in its multifaceted nature, as it needs to combine cognitive processes and clinical decision-making within various physiotherapy disciplines.

According to Cencetti et al. (2023), this variation in CR application creates challenges for educators in teaching and assessing CR development. A study by Talberg, Camroodien-Surve and Amosun (2021) revealed that students’ understanding of CR often differs from that of their clinical educators (CEs); these differences may result in students missing crucial cues during the CR process, and such misalignments could potentially impede learning outcomes. Talberg et al. (2021) argue that the diverse backgrounds of students and educators contribute to different interpretations of CR. This could potentially impact learning outcomes when students and educators lack a shared understanding of CR principles and lead to students focusing on incorrect or less relevant aspects of CR. Alignment would be important to ensure learning outcomes are met by ensuring learning activities and assessments support the development of CR.

Wainwright and Gwyer (2017) emphasised that while existing literature provides a foundation for the educational research agenda on CR, there is still a need to establish a shared understanding of CR. Singh et al. (2021) assert that for CR learning to be evident in the curriculum, programmes must commit to a shared understanding that establishes a common ground, facilitating effective interactions between educators and students. At the University of the Western Cape, CR is not taught as a standalone module. Instead, lecturers and CEs across disciplines in physiotherapy employ various strategies they believe will enhance students’ CR abilities in the classroom and during clinical placements. These clinical placements, coupled with weekly CE support sessions, provide a somewhat fragmented approach to CR development, which may lack consistency and alignment between educators and students.

Our study therefore aimed to use a qualitative exploratory descriptive design to explore how physiotherapy students and educators (both lecturers and CEs) of the physiotherapy department at the University of the Western Cape understand CR. By exploring their perspectives, this research seeks to identify any potential gaps in their shared understanding of CR and examine ways to better align their viewpoints.

## Research methods and design

Adopting an interpretivist paradigm, our study employed a qualitative exploratory descriptive design to investigate the varied meanings and understandings of CR among key stakeholders (Creswell 2015), including undergraduate physiotherapy students, lecturers, and CEs. The methodological approach and design allowed for all perspectives to be considered in the current context.

### Data collection

Data were collected via in-depth interviews (Online Appendix 1, Table 1-A1) to understand the perceptions of the participants (Mays & Pope 2000). In-depth interviews bring meaning to complicated social issues that are relevant and allow for the production of information on the participants’ experiences (DiCicco-Bloom & Crabtree 2006). Interviews were conducted in English as the authors sought out detailed information on the CR process and the understanding of clinical reasoning from the students, CEs, and lecturers. Interview guides (Online Appendix 1, Table 1-A1) were used to collect the data, ensuring a consistent yet flexible approach to questioning across all participants. Broad, open-ended questions were designed to explore the participants’ understanding of CR in depth, while specific probes were used for clarification, exploring emerging ideas and encouraging participants to expand on their responses. Each interview lasted 45–60 min. Recording methods varied; online interviews used Zoom conferencing, while face-to-face interviews employed a cell phone audio recorder. All lecturer interviews were conducted face-to-face at locations chosen by the interviewees. Student and CE interviews were either face-to-face at participant-selected locations or online via Zoom, based on individual preferences.

### Participants and recruitment procedures

Participants included registered third- and fourth-year physiotherapy students, CEs, and lecturers who were employed at the university at the time of the study. Both CEs and lecturers were involved in the students’ clinical education. Purposive sampling was used to ensure diversity, as this sampling method involves identifying and selecting a diversity of individuals that have an interest in the same phenomenon (Etikan Musa & Alkassim 2016; Mays & Pope 2000); therefore, all students registered for the clinical practice module and all employed lecturers and CEs were invited to participate in the study. The final sample size included participants who accepted the invitation to participate. These included physiotherapy students (*n* = 27), CEs (*n* = 8), and lecturers (*n* = 10). Informed consent was obtained from all participants.

This research was undertaken by J.H. and D.H. as part of their master’s and PhD degrees, respectively. Both led participant recruitment and data collection, and they are researchers and staff members within the physiotherapy department where data collection and participant recruitment took place. J.H. was employed as a CE at the time the study was conducted, and D.H. was a lecturer. This meant that they had direct contact with the students either in the classroom or the clinical setting and were colleagues of the CEs and lecturers who were interviewed.

A research assistant conducted 8 out of the 10 lecturer interviews; D.H. (one of the primary authors) was a colleague of the lecturer participants. Due to unavoidable scheduling conflicts, D.H. conducted the remaining two lecturer interviews. For the student interviews, D.H. interviewed 18 of the 27 student participants, while J.H. interviewed 9. All CE interviews were conducted by J.H. despite being employed as a CE at the time of data collection.

### Data management and analysis

Data analysis was carried out by D.H. and J.H. Interviews were transcribed verbatim by an independent transcriber. Braun and Clarke’s (2019, 2021) six-phase thematic analysis was used to analyse the data. This method of data analysis allows for patterns to emerge from the data itself (Braun & Clarke 2019). It also emphasises organic theme development (Braun & Clarke 2021). All audio recordings were listened to and transcripts were read multiple times by both authors. Segments of text were highlighted, and codes were assigned to these highlighted texts, directly from the interview data in the transcripts. The codes were then reviewed, merging repetitive codes and discarding those that did not make sense. The codes were then collated into themes. The themes were defined and named. The results are presented below, using quotes to bring meaning to the themes.

Trustworthiness was enhanced using the criteria of Guba, which included credibility, transferability, dependability, and confirmability (Shenton 2004). To ensure credibility, eight participants were invited to view and comment on the accuracy of the authors’ interpretations of the data received from them, and no changes were suggested. Transferability was ensured by providing a detailed (thick) description of the study processes, allowing for applicability of the findings to other research contexts, circumstances, and situations (Stahl & King 2020). The process of the study was documented in detail, thereby ensuring dependability, which would allow future researchers to repeat the study. In addition, the coded data were regularly discussed with study supervisors to assist in verifying the interpretation of data and refining emerging themes. Confirmability was established by making use of an audit trail highlighting every step of the data analysis, accurately portraying the participants’ responses, and in-depth descriptions of the study’s methodologies were given to allow scrutiny of the integrity of the research findings. The research design, study participants and processes are outlined, data analysis explained and codes, themes and corresponding quotes are shared. The authors’ positions as insiders in the department were acknowledged and managed. Both authors acknowledged their link to the students, CEs, and lecturers and constantly discussed data interpretations with the study supervisors to minimise bias (Bourke 2014).

### Ethical considerations

The ethics reference number for the data collected by D.H. was HS17/5/18, and for the data collected by J.H., it was HS19/9/20. Both projects were approved by the Humanities and Social Science Research Ethics Committee of the University of the Western Cape. Participants were provided with information sheets, which explained what the study entailed as well as any potential risks and benefits of their participation. All participants gave written informed consent in either hard copy or electronic format to both the authors. All transcripts were coded to ensure anonymity, and privacy was ensured by storing data in the Google Drive attached to each author’s student account, which is password-protected. All participants were made aware that participation was voluntary, would not affect their academic standing or professional relationships, and they could exit the study at any time without penalty.

## Results

The overall sample included 10 lecturers, 8 CEs, and 27 undergraduate physiotherapy students. The demographics related to the participants are displayed in Table 1, which highlights the gender distribution, clinical and teaching experience of the educators, and the clinical exposure of the students.

**TABLE 1:** Demographic characteristics of study participants.

Study participant group	*n*		Clinical experience	Teaching experience and clinical teaching experience (years)
	Female	Male		
Lecturer	6	4	7.8 years†	8.9 years†
Clinical educator	6	2	11.5 years†	5 years†
Student	25	2	1 year‡ 2 years§	N/A

N/A, not applicable.

† , years (mean);

‡ , *n* = 7;

§ , *n* = 20.

Participants in our study described clinical reasoning as a multifaceted and dynamic process that is integral to physiotherapy practice. Their responses revealed an understanding that encompasses cognitive aspects of decision-making, the emphasis of the importance of integrating theoretical knowledge into practical skills, and the inclusion of the patient. The analysis revealed three key themes that encapsulate the participants’ understanding of clinical reasoning: cognitive processes, evidence-based practice, and clinical approach. All themes, codes, and quotes are outlined in Online Appendix 1, Table 2-A1. The themes, their codes, a description of the meaning of the code, and an explanatory quote are described in the following sections.

### Theme 1: Cognitive processes

The theme of cognitive processes emerged from codes that included knowledge, the ability to collect and connect information regarding the patient, decision-making, problem-solving, hypothesis generation, and justification of action.

The participants described *knowledge* as the ability to draw on existing knowledge, such as lectures, notes, and textbooks, to inform patient management. A CE explained it this way: ‘… it basically starts with understanding your anatomy and your physiology and that ties together what you see and what you eventually will assess’ (participant 7, female, clinical educator).

*Collecting information* was identified as an essential skill, involving the gathering of details from various sources such as patients, their medical folders, family members, or other healthcare professionals. A student highlighted this process: ‘… the information you have gathered from your patients and from the file and being able to use that to focus your assessment and treatment of your patient …’ (participant 15, female, student). Once information is collected, the next step is *connecting information* to form a comprehensive understanding of the patient’s condition. A lecturer explained: ‘They’ve got to know something in order to say well based on what I know here, and what I found here, I can then link the two and say this is what I need to do next’. (participant 1, female, lecturer).

*Problem-solving* was another cognitive process frequently mentioned, with participants describing it as the ability to navigate through patient-centred challenges. A student noted: ‘… it is the way that you get to the answer of a problem that a patient presents …’ (participant 18, female, student).

Only lecturers and CEs understood CR as involving hypothesis generation. This was described as integrating findings from various sources to create a coherent picture of the patient’s condition. As one lecturer noted: ‘So it is integrating findings from different sources, from what you see and from what you know and from what the patient tells you and trying to form a coherent picture’ (participant 10, female, lecturer). A CE added:

‘You see how everything fits together and you get the bigger picture of “okay so this is what’s wrong” and you already automatically know how you’re going to approach this problem …’ (participant 1, female, clinical educator)

Lecturers and CEs therefore had a greater focus on hypothetico-deductive generation as their understanding of CR; this shows their sophistication and experience, as CR is a combination of isolated processes that include collecting and connecting information.

In contrast, *justification of action* was a key component identified by students as central to their understanding of CR. For students, this involved articulating the rationale behind their decisions and interventions. As one student explained, ‘…clinical reasoning for me is basically why are you testing muscle power, so why are you doing what you are doing?’ (participant 22, female, student). Interestingly, this was not emphasised by the lecturers or CEs, reflecting a divergence in perspectives.

### Theme 2: Evidence-based practice

Two key codes supported the theme of evidence-based practice: the ability to *apply theory to their practice* and the *use of information gathered* about the patient. Many lecturers defined CR as the ability to apply classroom knowledge to real-world applications, emphasising the importance of translating theory into practice. One lecturer explained:

‘So clinical reasoning for me is if you can apply what you’ve learnt in class better as being in a practical or a theoretical lecture, if you can apply it to a patient, the real scenario, you go out to the hospital and you decide okay, this is now the best technique.’ (participant 2, female, lecturer)

The second aspect of evidence-based practice, using patient information to make decisions, was highlighted by a student:

‘My understanding of clinical reasoning is for example when they are in a clinical placement and then you get a patient and then the patient starts explaining to you the signs and symptoms and then you use those signs and symptoms to diagnose the patient and then plan the treatment.’ (participant 7, female, student)

These insights underscore the dual focus of CR as both applying theoretical knowledge and integrating patient-specific information to inform decision-making.

### Theme 3: Clinical approach

The only code for this theme was *patient-focused*. Both the educators and the students viewed CR as the ability to make patient-centred decisions, emphasising the patient’s integral role in the process. This perspective is encapsulated by one lecturer, who explained:

‘Every day you are looking at the patients in a new way, and so for me clinical reasoning is that process of trying to come to an understanding of your relationship with the patient today, at this moment.’ (participant 6, male, lecturer)

A student explained it this way: ‘ … your thinking will vary from patient to patient because it’s all different situations, scenarios, etcetera’ (participant 11, female, student). This highlights the dynamic and evolving nature of CR, rooted in the immediate and individualised needs of the patient.

## Discussion

The study aimed to explore how physiotherapy students and educators understand CR to determine whether their perspectives align, particularly regarding a skill that is inherent in educators but still being developed in students. Three key themes emerged from the analysis: cognitive processes, evidence-based practice, and clinical approach. Examining the similarities and differences in how these themes manifested across different levels of expertise provides valuable insights to inform physiotherapy education and practice.

The participants in our study understood CR as a cognitive process. Furthermore, they focused on collecting information, connecting information and problem-solving. Students have a greater focus on justifying the reasons for their choices, whereas the lecturers and CEs focus on hypothesis generation, highlighting a clear gap in the reasoning sophistication between students and educators. Similarly, Koufidis et al. (2021) identified three main conceptualisations of CR, one of which included reasoning as a cognitive activity. Koufidis et al. (2021) further explain that cognitive activity includes problem-solving, which, when broken down, is to develop provisional hypotheses. This determined how they collected data, and then they would either negate or confirm the data. The students also saw CR as each of its various isolated parts and the educators more as a holistic process. This was similar to Doody and McAteer (2002), who investigated the CR process of physiotherapists and identified differences in CR between experts and novices. Doody and McAteer (2002) highlighted the following aspects of patient treatment in a musculoskeletal setting: collecting data about the patient, hypothesis generation, evaluating the data, hypothesis evaluation, and then treatment decision. They found that these steps in the CR process were not always possible for novices; after evaluating the data, novices could not necessarily go to hypothesis evaluation. Experts, however, were always able to make a judgement. This difference in how novices and experts operationalised CR highlights a gap.

The gap between expert (lecturer and CE) and novice (student) understanding of CR therefore presents significant challenges to effective teaching and learning, as it impacts the scaffolding required for students to progress towards expert-level reasoning. Ruczynski et al. (2022) highlight how educators may struggle to comprehend how challenging certain tasks are for the students, potentially leading to insufficient contextual details and assumptions about the students’ knowledge. This gap between the expert and novice understanding of CR is evident, where the educators and students in this study did have different views of how they understood it. As Talberg et al. (2021) suggest, CEs should aim to construct learning opportunities that describe the multidimensional nature of CR, which would both support students and scaffold the development of their CR as they progress through their clinical years.

The student participants highlighted the importance of justifying their actions, which aligns with the emphasis on justification in the clinical examination rubric used for assessment. This focus on justification was not mentioned by lecturers or CEs, suggesting a potential misalignment in perspectives. This misalignment indicates a need for educators to better understand students’ viewpoints and bridge the gap between teaching and learning approaches and assessment expectations. As Durak et al. (2007:170) observed, ‘examinations drive learning’, highlighting how assessment can transcend from an evaluative tool to an active learning intervention. This indicates the importance of designing rubrics and assessment tools that reflect CR as a holistic process, integrating all its components rather than isolating individual steps.

Lecturers further emphasised the importance of applying theory to practice as their understanding of CR, reflecting their primary responsibility for delivering theoretical content. Case-based learning (CBL) has been recognised as an effective approach to bridge the theory-practice gap, fostering the development of CR skills by immersing students in scenarios that mirror real-world practice (Thistlethwaite et al. 2012). Case studies would typically incorporate the patient history, clinical examination findings, and investigation results (Klemenc-Ketis, Cagran & Dinevski 2018). Modi, Anshu and Singh (2015) advocate for the use of case-based discussions as an ideal strategy for teaching CR skills – whether conducted independently or collaboratively (Macartney, Cooper & Namasivayam 2021). Additionally, Ravat, Barnard-Ashton and Keller (2021) assert that group case-based discussions not only cultivate critical thinking but also improve communication and collaboration skills, which further enhance the learning experience.

Implications for practice therefore include creating environments where expectations are clearly defined, shared goals are established and opportunities exist to acknowledge and align the students’ and educator’s perspectives. Meyer, Louw and Ernstzen (2017) suggest that explicitly addressing assumptions and fostering shared understanding among students and educators can help redefine traditional roles in the teaching and learning relationship between educators and students. Design rubrics that explicitly value both novice priorities (clear justification of actions) and expert processes (hypothesis generation). By intentionally designing rich, contextualised CBL activities in the classroom, educators can create a foundation that CEs can build upon, ensuring students can transfer their learning seamlessly to real patient scenarios on the clinical platform. However, CEs play an important role in further supporting this learning by educating students on the types of reasoning they use to facilitate CR and intentionally make their own thinking processes visible during clinical teaching sessions (Talberg et al. 2021). By aligning classroom CBL with on-the-ground clinical teaching, educators can reinforce the application of CR, ensuring that students are better prepared for real-world patient care.

In terms of further research, the authors propose critical investigations that address key pedagogical challenges in physiotherapy education. Specifically, research is recommended to explore optimal strategies for implementing CBL, with particular emphasis on contexts with limited resources. Additionally, future studies should focus on developing comprehensive assessment methodologies for CR skills, with the aim of bridging the understanding gaps between educators and students. These research directions are necessary for advancing CR pedagogy and ensuring more aligned, effective educational approaches in physiotherapy training.

The authors acknowledge a limitation of this study was the potential influence of power dynamics during data collection, as the authors were insiders in the department with established relationships with the participants. Strategies to mitigate these dynamics included using a research assistant for most of the lecturer interviews, offering participants a choice of interview mode and location, and ensuring confidentiality and regular debriefing sessions with study supervisors about data interpretation. Despite these limitations, the consistency in themes across participant groups strengthens the trustworthiness of the findings.

## Conclusion

When students and educators in the same department share a common understanding of CR and its associated tasks, it creates intentional opportunities for growth. This shared perspective allows all stakeholders to apply their knowledge of CR effectively in diverse clinical settings and across multiple disciplines of physiotherapy. The study reveals both opportunities and challenges in developing CR competency in physiotherapy education. While there is fundamental alignment in understanding CR importance, the different emphasis placed on CR components by the students versus educators suggests the need for more intentional educational approaches. The authors would like to propose the need for structured dialogue between stakeholders about the CR process in order to impact the effect of teaching and learning outcomes. By ensuring all stakeholders are focused on the same elements of CR, we can enhance the development of the skill in future physiotherapists. In addition, balanced assessment approaches must be identified, which acknowledge both foundational and advanced CR skills.
